# Reclaiming the Stroop Effect Back From Control to Input-Driven Attention and Perception

**DOI:** 10.3389/fpsyg.2019.01683

**Published:** 2019-08-02

**Authors:** Daniel Algom, Eran Chajut

**Affiliations:** ^1^ School of Psychological Sciences, Tel Aviv University, Tel Aviv, Israel; ^2^ Department of Education and Psychology, Open University of Israel, Ra’anana, Israel

**Keywords:** Stroop, control, conflict, salience, congruity, contingency

## Abstract

According to a growing consensus, the Stroop effect is understood as a phenomenon of conflict and cognitive control. A tidal wave of recent research alleges that incongruent Stroop stimuli generate conflict, which is then managed and resolved by top-down cognitive control. We argue otherwise: control studies fail to account for major Stroop results obtained over a century-long history of research. We list some of the most compelling developments and show that no control account can serve as a viable explanation for major Stroop phenomena and that there exist more parsimonious explanations for other Stroop related phenomena. Against a wealth of studies and emerging consensus, we posit that *data-driven selective attention* best accounts for the gamut of existing Stroop results. The case for data-driven attention is not new: a mere twenty-five years ago, the Stroop effect was considered “the gold standard” of *attention* (MacLeod, 1992). We identify four pitfalls plaguing conflict monitoring and control studies of the Stroop effect and show that the notion of top-down control is gratuitous. Looking at the Stroop effect from a historical perspective, we argue that the recent paradigm change from stimulus-driven selective attention to control is unwarranted. Applying Occam’s razor, the effects marshaled in support of the control view are better explained by a selectivity of attention account. Moreover, many Stroop results, ignored in the control literature, are inconsistent with any control account of the effect.

Everyday functioning requires a modicum of ability to attend selectively to the relevant feature of objects, excluding irrelevant or distracting features. In the absence of this ability, one cannot concentrate on texting a friend in the cafeteria, listening to a presentation in class, or negotiating the traffic when driving or walking. Facility at isolating the task-relevant attribute is indispensable for adaptation and survival. The Stroop effect (Stroop, 1935) assays this vital mental faculty. In fact, the Stroop effect is psychology’s oldest and still most popular tool for assessing the ability at focusing exclusively on the attribute of interest in the object (Eidels et al., 2010). In Stroop’s (1935) original setup, the objects were color words printed in color, and the relevant attribute for responding was the color (while ignoring the carrier word). To gauge the influence of the task-irrelevant words, the Stroop effect is defined as the difference in color-naming performance between congruent (the word naming its color such as RED in red, with the former indicating the word and the latter the color) and incongruent (word and color conflict, such as RED in green) stimuli. Better performance with congruent than with incongruent stimuli shows that people paid attention to the task-irrelevant words, thereby compromising exclusive focus on the print colors. Had people focused exclusively on the target color, no word dependent difference in color naming (=Stroop effect) would have emerged. A century after Stroop’s landmark study, the effect bearing his name continues to fascinate researchers, sustaining an ever growing amount of studies. Despite the vast literature, the effect has eluded a consensual theoretical resolution.

## A Bit of History

The Stroop effect boasts a convoluted history. In the first period, between 1935 and 1964, the effect attracted little interest and was discussed as a learning phenomenon (MacLeod, 1992). In Stevens’ (1951) celebrated handbook, there is but a single passing reference to Stroop in a chapter on learning and retention. After 1964, the theoretical interpretation of the effect changed dramatically to one of attention (Klein, 1964; Jensen and Rohwer, 1966). The number of publications rose quickly, and the pace shows no signs of abating to date. The new construal of the Stroop effect occurred contemporaneously with the advent of the cognitive paradigm in psychology. The trend of accommodating attention peaked in the last decade of the Twentieth century. Colin M. MacLeod, author of the definitive review (MacLeod, 1991), called the Stroop effect “one of the benchmark measures of *attention*” (MacLeod, 1992, p. 12).

However, the dominant conceptual framing of the Stroop effect changed yet again at around the turn of the twenty-first century. The new approach centered on the notions of “conflict” and “control.” It was actually the latter term that was first popularized by Posner and his associates (e.g., Posner and Petersen, 1990; Posner and Raichle, 1994; see also, Petersen and Posner, 2012). These authors conceived performance in the Stroop task to be under “executive control” (Fan et al., 2002, p. 341) or simply as an “executive function” (Petersen and Posner, 2012, p. 73) under the control of well localized brain loci (in particular, the anterior cingulate system). Of course, it would be absurd to deny brain control of whatever we do, but assuming minute monitoring and very-small-scale response adjustments *via* central command ignores the influence of input-driven bottom-up processes. An all-engulfing central control view would still need to explain the ways and means of top-down penetration of Stroop performance on such a fine-grain scale. For all his efforts at identification of brain loci for cognitive functions, Posner was aware of the fact that these associations did not amount to a (Stroop) theory, to wit, “much needs to be learned about the *mechanisms*” used by the “executive system” (Posner and Raichle, 1994, p. 174, emphasis added). Subsequent development of the control view claimed to identify such a specific top-down mechanism – conflict monitoring and management – which governs Stroop performance. This novel theory of the Stroop effect rests on the original observation by Posner and Raichle, 1994 that “the anterior cingulate system is more active during trials of the Stroop task in which conflict exists than during trials in which it does not” (p. 171). However, more recent research increasingly questions an exclusive connection between enhanced activity of the anterior cingulate system and conflict (e.g., Steinhauser and Hiibner, 2009; Grinband et al., 2011a; Levin and Tzelgov, 2016; see also again, Posner and Raichle, 1994).

Conflict monitoring theory (Botvinick et al., 2001) proposes that performance in the Stroop task is governed by central control, which adjusts the attention allocated to the target color on a trial-to-trial basis. In particular, Stroop-incongruent stimuli generate a large amount of conflict (due to the mismatch between the color and the word). This conflict, in turn, invites increased control, which subsequently reduces the attention allocated to the task-irrelevant word. It is difficult to overstate the grip on current research of the control account. The fad of conflict monitoring and control is unprecedented within the Stroop milieu; following Schmidt’s (2019) observation, the first few articles published between 1998 and 2004 now combine for over 30,000 citations in the literature (e.g., Carter et al., 1998; Botvinick et al., 1999, 2001, 2004; MacDonald et al., 2000; Miller and Cohen, 2001; Kerns et al., 2004; see Schmidt, 2019, for an extensive bibliography). The upshot is, the Stroop effect has been appropriated from being an index of input-driven selective attention to a tool for generating conflict and measuring control.

## Goal of The Present Review

We believe that the recent paradigm shift in the construal of the Stroop effect is unwarranted. Our goal in this review is to show, against a wealth of recent studies and emerging consensus, that there is in fact no compelling evidence for control or top-down influence in the Stroop effect. Certainly, the term “top-down” is used in a variety of ways in different domains of cognitive psychology (see Firestone and Scholl, 2016). Within the Stroop milieu, “top-down” influence is currently conceived as an overall strategy, which is typically determined in advance. It is exercised through control and results in adaptation to conflict. It is this meaning of “top-down” influence that we challenge as a valid theory of the Stroop effect.

We are not alone in challenging the conflict monitoring account. In the face of an overwhelming literature, James Schmidt has mounted a powerful attack on the psychological reality of conflict monitoring and control, dubbing them repeatedly “an illusion” (e.g., Schmidt et al., 2015, 2018). In two comprehensive reviews, Schmidt concluded that data-driven explanations (e.g., biased learning and memory) provide a sufficient account of the findings subsumed under the conflict monitoring and control (Schmidt, 2013, 2019; see also, Schmidt and Besner, 2008; Schmidt, 2016a,b). Notably, Schmidt’s alternative explanation does not appeal to the notion of conflict and control. Schmidt addresses in admirable detail the various biases lurking in major control studies and concludes that those biases compromise their validity as well as the attendant explanation in terms of conflict and control. Given Schmidt’s contribution and the availability of further comprehensive reviews of the control literature (e.g., Egner, 2008, 2014; Bugg and Chanani, 2011; Bugg and Crump, 2012; Bugg and Hutchison, 2013; Bugg, 2014; Abrahamse et al., 2016; Cohen-Shikora et al., in press), we eschew another general review. Instead, the present article is a theoretical critique of the control account, one rooted in bona fide Stroop literature.

The present review takes the neglect of basic Stroop results in control studies as a point of departure and expands the analysis to show that conflict monitoring and control cannot serve as a viable theory of the Stroop effect. As we recounted, the Stroop effect boasts a long and rich history (rapidly approaching the century mark), but large chunks of this research are ignored in the control literature. We show that factoring in basic findings of proper Stroop research challenges the validity of any theory of conflict monitoring and control.

## The Structure of the Review

To anticipate the development, we first state in a concise fashion our main argument. Four pitfalls plaguing control studies of the Stroop effect are then pinpointed. We follow by discussing each point in detail. These discussions, informed by basic Stroop literature, form the backbone of the paper. The understanding that conflict or control accounts do not comprise a viable candidate explanation of the Stroop effect is stated in the section “Conclusion.”

## The Main Argument: What is and what is not Explained by Conflict and Control?

Very succinctly, the conflict monitoring account proposes that attention is dynamically allocated to either the target (color) or the distractor (color word) *via* central control. Each time high conflict is met (by a Stroop-incongruent stimulus), control is engaged to enhance focus on the target. This amplified control is relaxed when high conflict is not experienced (by a Stroop-congruent stimulus). Of the wide range of Stroop-related phenomena (see, e.g., MacLeod, 1991; Melara and Algom, 2003, or Sabri et al., 2001, for reviews), the evidence for the conflict monitoring account is based almost exclusively on two effects: the proportion congruent (PC) effect and the sequential effect known as the Gratton effect (Gratton et al., 1992).

## What is Explained by Conflict Monitoring and Control?

The PC effect is the observation that the Stroop effect is smaller when there are a disproportionately large number of incongruent stimuli in the set. For example, the Stroop effect is smaller when the stimulus ensemble includes 80% incongruent stimuli (hence 20% congruent stimuli) than when the ensemble includes 20% incongruent stimuli (hence 80% congruent stimuli). The conflict monitoring account provides a ready explanation for this modulation of the Stroop effect: Participants experience a great deal of conflict in the mostly incongruent set, a condition that is bound to summon strong central control. The enhanced control, in turn, results in focused attention to the target attribute. The task-irrelevant word is less attended, and the net result is a small Stroop effect. Therefore, the greater the number of incongruent stimuli, the smaller the Stroop effect.

The Gratton effect is the observation that the (color) response to an incongruent stimulus that follows an incongruent stimulus is faster than the response to an incongruent stimulus that does not follow an incongruent stimulus (i.e., it is preceded by a congruent stimulus). The same explanation is offered by the conflict account, now on a smaller scale. After experiencing conflict on trial *n*−1, control is invited to exert its influence, so that its salutary effect is observed on trial *n*. In other words, due to enhanced control, the participant adapts to conflict and maximizes the ability to ignore the task-irrelevant word.

In summary, this new account provides reasonably straightforward explanations for these two effects in terms of conflict, control, and conflict adaptation. There is a pitfall, though: Much simpler explanations are available based on properties of the data at hand. We discuss these stimulus-driven explanations and show that they are to be favored over control on grounds of both parsimony and general applicability.

## What is not Explained by Conflict Monitoring and Control?

Whereas alternative explanations exist for the PC and the Gratton effects (Schmidt, 2019), conflict monitoring and control theory have real difficulty explaining the following Stroop finding. Presenting the *same* number of incongruent stimuli can result in a large Stroop effect, a zero Stroop effect, or a reverse Stroop effect (where colors intrude on word naming more than vice versa). The trifle stimulus manipulation that produces these diverse outcomes is slight changes in the relative salience of the color and the word components of the stimulus. It is important to note that the changes of salience are so slight that the words remain eminently legible and the colors similarly remain eminently identifiable under all the conditions. These findings are devastating for the control account (e.g., Garner and Felfoldy, 1970; Garner, 1974; Pomerantz, 1983; Melara and Mounts, 1993; Algom et al., 1996; Melara and Algom, 2003; Algom and Fitousi, 2016). Presumably the same amount of conflict is experienced, yet performance changes dramatically regardless of “conflict.”

Quite apart from these observations, portions of the Stroop literature contain studies in which presentation of Stroop stimuli – i.e., conflict generating stimuli – does not yield a Stroop effect (e.g., Flowers et al., 1979; McClain, 1983a,b; Glaser and Glaser, 1989). Again, no control explanation is able to account for such results. In general, control theory is unable to explain variation in Stroop results when the amount of conflict is held constant.

A further observation is arguably fatal for control theory: congruent stimuli produce Stroop facilitation (faster color naming to congruent than to neutral stimuli) just as incongruent stimuli produce Stroop interference (faster color naming to neutral than to incongruent stimuli), and the Stroop effect entails *both*, i.e., the effect is not solely interference. Thus, participants respond “red” faster to the word RED in red than to the word TABLE in red, a result called facilitation, and the Stroop effect is sometimes generated wholly or mostly by facilitation rather than by interference (Brown, 2011; Eidels, 2012). The faster RTs to congruent than to neutral stimuli – Stroop facilitation – is not a transient or ephemeral result; it is a systematic effect (as much as Stroop interference), and conflict monitoring theory seems unable to account for a Stroop effect produced by facilitation. Finally, control theory faces difficulty in accounting for Stroop’s original results (Stroop, 1935). In Stroop’s experimental condition, all of the stimuli were incongruent, so that control was presumably very strong. Conflict monitoring theory predicts a small Stroop effect (interference). In sharp contrast to this prediction, Stroop recorded what is arguably the largest Stroop effect in the literature.

In the remainder of the review, we expand on all the above points. We show that effects attributed to central top-down control are actually changes in the stimulus input; the effects are well captured by input-driven attention or its failure. Next, we identify four pitfalls lurking in studies performed under the control approach.

## Four Pitfalls in Control Studies of the Stroop Effect

**First**, arguably the most severe pitfall is that key term of “conflict” in the “conflict-generated-control” approach is vague and imprecise. The problem is already apparent in the widely cited study of Botvinick et al. (2001), a pioneering undertaking in the field. The notions of “conflict monitoring” and “control” are thoroughly discussed, but what is missing from the text is a clear, unambiguous *theoretical* definition of the key term of “conflict.” Monitoring is rightly showcased as the new development (the added component to the computational model of Cohen et al., 1990, or that of Cohen and Huston, 1994), but what is being monitored is underdefined. In lieu of a theoretical definition, Botvinick et al. (2001) ponder how “conflict might be *measured*” or “*operationally* defined” (p. 630; emphases added). For a tool, the authors elected to use Hopfield’s (1982) measure of “energy” in a recurrent neural network to indicate the level of conflict; in words, “conflict” is conceived as “the simultaneous activation of incompatible representations … e.g., representations of alternate responses” (Botvinick et al., 2001, p. 630). This definition is imprecise as is. In particular, the notion of “incompatible representations” is left hopelessly ambiguous.

To understand the cost of the ambiguity, consider the following critical question. Does “conflict” and “incompatible representations” apply only to *logically* contradictory responses (hence, to truly incompatible responses) or to all possible responses to multidimensional stimuli? To render the question more concrete: Is a circle in green and the word RED in green *both* conflict stimuli? With the first stimulus, there is no logical or semantic conflict (or agreement) between color and shape. There cannot be congruent and incongruent cases with stimuli composed of color and shape – a green circle is neither more nor less congruent or incongruent than say a blue rectangle. The Stroop effect cannot be calculated for such stimuli simply because the Stroop effect is defined by the difference between congruent and incongruent cases. A certain shape and a certain color cannot be in conflict because neither excludes the other; the responses to the shape and the color of a green apple are never incompatible. By contrast, the second stimulus *is* a Stroop stimulus: The word and the color can match (=congruent stimulus) or conflict (=incongruent stimulus). An incongruent Stroop stimulus is a genuine conflict stimulus because the response to the word excludes the response to the color. The responses to the word and to the color are inescapably incompatible. Conversely, for the congruent Stroop stimulus, RED in red, the responses to the word and the color do not compete with one another as they are the very same single response. Because the responses are compatible (not incompatible), congruent Stroop stimuli are free of conflict. Considering the Botvinick et al. (2001) model, the approach called “conflict monitoring and control” does not appreciate or recognize the qualitative difference between Stroop or conflict stimuli, on the one hand, and non-Stroop or non-conflict stimuli, on the other hand. Adverse consequences ensue for theory and research alike.

In the computational model of Botvinick et al. (2001), virtually all multidimensional stimuli are conflict stimuli, i.e., Stroop-congruent stimuli such as RED in red and non-Stroop stimuli such as a green apple all are conflict stimuli. This feature alone defies common sense and violates fundamental laws of logic. For common sense, to maintain the absurd thesis that RED in red produces conflict – when both components agree, support, and converge on the same single response – is tantamount to leaving the notion of conflict void of meaning. For logic, to discount the structural difference between the Stroop-incongruent stimulus, RED in green, and the non-Stroop stimulus, green apple, means ignoring the basic law of non-contradiction. For RED in green, the possible responses (red, green) cannot both be true (for that ink color), so that the responses are mutually exclusive. By contrast, for a green apple, the possible responses (green for color and apple for shape) can both be true at the same time, so that the responses are not mutually exclusive. In logic, the truth-functionally compound statements (e.g., Copi, 2015) that are (or that can be) associated with RED in green and with a green apple are fundamentally different. Again, this difference is ignored in the model. Thus, Botvinick et al. (2001) affirm in their text that on “*incongruent* trials … the intersection of … two pathways … causes *conflict*” (p. 631, emphasis added), but this tells only part of the story; in their model, congruent trials also generate (less) conflict.

To recap, the Botvinick et al. model holds that Stroop-congruent stimuli, Stroop-incongruent stimuli, non-Stroop stimuli, all produce conflict to a different degree. The difference is merely quantitative. By contrast, common sense, logic, and insights based on a century of Stroop research hold that (1) incongruent stimuli entail conflict, (2) non-Stroop and neutral stimuli lack the quality of conflict (conflict is orthogonal to such stimuli), and (3) congruent stimuli are free of conflict. Although computationally elegant and manageable (and parsimonious), the idea that Stroop-congruent (and non-Stroop) stimuli cause conflict is conceptually untenable.

The tenuous relation in the model between Stroop-congruity and conflict came to the fore in subsequent extensions of the model, which also included errors (Yeung et al., 2004, 2011; Yeung and Nieuwenhuis, 2009). The extended versions each used a different implementation of the model, which, in turn, affected the Congruity-Conflict predictions to the extent that it was questioned “whether a single unified model of conflict monitoring exists” (Grinband et al., 2011b, p. 321). In the more recent version of Yeung et al. (2011), “conflict” is conceived as enhanced anterior cingulate activity that can result from a large variety of sources, including sensory noise, attention fluctuation, and response bias – all of which can and often do “dwarf” congruity-related conflict. Maintaining that “conflict” corresponds to *any* unrelated sensorimotor activity (that affects RT) leads to the absurd idea that “conflict” exists even when detecting a simple one-dimensional signal with a *single* response option. This “diffuse definition” of conflict (if it is a definition in the first place) “trivializes” the concept of conflict, making it practically useless (Grinband et al., 2011b, pp. 321–322). In the final analysis, “conflict” in the Yeung et al. (2011) model is basically independent of congruity and is independent of response compatibility (see again, Grinband et al., 2011b); the notions of congruity and (in)compatibility that first motivated the Botvinick et al. (2001) effort are trivialized in later implementations of the model. As a result, the model is an ill-suited candidate theory of the Stroop effect.

We identify three fundamental problems with the Botvinick et al. (2001) approach (and its various offspring). First, as noted in Grinband et al. (2011b), conflict monitoring was never tested against the natural null hypothesis that enhanced anterior cingulate activity is associated with task general processes of perception, attention, and memory, rather than with conflict. When tested against this null hypothesis (Grinband et al., 2011a), no evidence for involvement of conflict (monitoring) was found beyond the generic effect of task engagement. The second fundamental problem is that the model couples a highly specific and richly developed concept from cognitive psychology to electrophysiological activity in a certain brain region – ignoring throughout the loaded ramifications of the concept within cognitive science and philosophy. Instead, the model (especially in recent implementations) stretches the notion of conflict beyond reasonable limits (the model might well have used “energy” or any other term to replace the increasingly debilitated “conflict”). The third fundamental problem concerns methodology, namely the scientific value and usefulness of the concepts of “conflict” and “control.” In the model, virtually any act of perception and cognition is marked by conflict. Conflict is lurking beneath such quotidian actions as reading familiar words, deciding between independent non-opposing alternatives, or just responding to any stimulus in an unspecified manner. However, if everything is conflict, then conflict becomes an empty, useless concept. A useful scientific definition should specify not only what is included, but also what is excluded.

Finally, inconsistent with the computational model discussed, the majority of Stroop studies subsumed under the control idea do place conflict quite naturally in Stroop-incongruent stimuli. As a rule, Stroop-incongruent trials are defined as “conflict stimuli,” implying that Stroop-congruent stimuli are free of conflict. This binary conception is the dominant and accepted view in large portions of the control literature. The terms “incongruent stimuli” and “conflict stimuli” are used interchangeably in the control literature (e.g., see the titles of Bugg and Smallwood, 2016, or of Mayr et al., 2003). We reiterate, the term “conflicting stimuli” implies non-conflicting stimuli (i.e., congruent or neutral stimuli), and this distinction actually informs much discussion of the Stroop effect in the control literature. Nevertheless, we return to discuss the implications of basic Stroop findings for the continuum conception entailed in the computational model and show that “conflict” and “control” are superfluous to an explanation of the varieties of Stroop effects.

**Second**, in the “conflict-generated-control” approach, parallel processing or cross-talk is typically tailored to result in interference. However, a cross-talk can also result in facilitation and in a gain to performance (MacLeod, 1991; MacLeod and MacDonald, 2000; Roelofs, 2010). Again, the prime example in the control literature of cross-talk produced *interference* is the Stroop effect. However, the Stroop effect is not solely interference; it is also facilitation. Stroop effects attributed to interference may well be those of facilitation. In the absence of partitioning the effect into interference and facilitation, a partition that is rarely done in control studies, one cannot decide the source. Without appropriate measurement, the Stroop effect cannot serve as arbiter of conflict.

Arguably, too, the notion of a Stroop effect produced by facilitation is anathema to the conflict-control approach (e.g., Lindsay and Jacoby, 1994; Brown, 2011; Eidels, 2012). After all, conflict is supposed to generate interference. However, if the same Stroop presentation systematically generates facilitation (rather than conflict and interference), the notion of enhanced control summoned by conflict is called into question.

**Third**, it is not completely clear where the conflict resides (e.g., Levin and Tzelgov, 2016). Does the conflict reside in the stimulus, i.e., impacting early input-driven processing, or does it mainly reside in the response? In the face of a certain level of ambiguity, most discussions and modeling efforts focus on late processing, close to the response. However, this conception can be challenged. Following Garner (Garner, 1962, 1970, 1974; Garner and Felfoldy, 1970; see also Melara and Algom, 2003; Algom and Fitousi, 2016), it is eminently possible that the conflict (mainly) resides in the stimulus. The problem is that authors within the control approach ignore the makeup of the stimulus. The perceptual properties of the Stroop stimulus – the physical features of the colors and the fonts used – are neglected. However, these basic perceptual properties can predict whether there will be a Stroop effect to begin with, as well as its direction (standard or reverse). For example, the relative perceptual salience of the presented color and word can determine if there is a Stroop effect, and, if there is, its magnitude (Garner, 1974; Melara and Mounts, 1993; Melara and Algom, 2003). Presenting Stroop stimuli does not *ipso-facto* guarantee that there is a Stroop effect! Depending on the perceptual properties of the stimuli, the *same* Stroop presentation can generate a Stroop effect, a zero Stroop effect, or a reverse Stroop effect (by which colors intrude on word reading more than vice versa; e.g., Pomerantz, 1983; Pomerantz and Pristach, 1989; Algom et al., 1996; Dishon-Berkovits and Algom, 2000). The upshot is, stimulus properties can determine the Stroop effect without need to engage any central control mechanism.

**Fourth**, the makeup of the stimulus is not the only data-driven mechanism governing the Stroop effect. Another data-driven influence on the Stroop effect is the correlation introduced over the experimental trials between the target colors and the task-irrelevant words. Because the Stroop *task* entails naming the color and because the Stroop *effect* measures the ability to attend selectively to the color, any color-word correlation introduced compromises exclusive attention to the color. A fair number of control experiments jeopardize the Stroop task by introducing just such a correlation between the relevant ink colors and the irrelevant words. The correlation makes the nominally irrelevant words predictive of the target color, so that attending to the word helps maximizing color performance. Inevitably, exclusive attention to the target colors is compromised. The original Stroop task as a measure of the selectivity of attention is disabled.

In several studies within the control approach (e.g., Bugg and Smallwood, 2016; Hutchison et al., 2016), the correlation between word and color over the experimental trials was created by the lopsided makeup of the block (for example, of a block of 10 trials, eight were congruent). In this case, the nominally irrelevant word largely predicts the target color. The situation is exacerbated by instructions that augment the actual correlation. For example, the participants are told that the majority (say, 80%) of the next block (of, say, 10 trials) will be congruent. The problem again is that this instruction and the attendant design already create a correlation between the nominally irrelevant words and the relevant colors, which is fatal for the selective attention tested (Dishon-Berkovits and Algom, 2000; Melara and Algom, 2003; Schmidt and Besner, 2008). Apart from the instructions, virtually all control studies entailed a word-color correlation by presenting (grossly) unequal number of congruent and incongruent stimuli. One must realize that imbalanced presentation of congruent and incongruent stimuli necessarily creates a correlation between the color and word components. Because (1) the Stroop effect measures (the failure of) selective attention to the color and (2) a color-word correlation diverts attention to the irrelevant word, a large Stroop effect is thereby created. Most important, this factor of correlation is stimulus dependent, i.e., it does not invite a central control mechanism to account for the Stroop results. All that is involved is simply the perception of correlation (Kareev, 1995a,b, 2000; Kareev et al., 1997).

We note that, in the control approach, providing advance information or biasing the probability of congruent and incongruent stimuli (by grossly imbalanced presentation) is legitimate. In this approach, these procedures are merely a means for generating conflict. What is not recognized though is that this way of generating conflict comes at the expense of compromising the meaning and the serviceability of the original Stroop test (as a tool of measuring selective attention). The manipulation is still called “Stroop,” but, in truth, it has almost nothing to do with the Stroop effect. It is thus hardly surprising that the Stroop effect itself is not calculated or is rendered marginal in a fair number of studies within the control approach (e.g., Hutchison et al., 2016; Kleiman et al., 2016; see also, Wegner and Erber, 1992; Wegner et al., 1993, on the use of the Stroop *task* without the calculation of the Stroop *effect* in “mental control”).

## Resolving the Pitfalls within Bona Fide Stroop Research

We proceed by elucidating the problems mentioned, benefiting from the results and insights obtained within Stroop research proper. To anticipate, resolution within genuine Stroop research shows that the notion of control is simply gratuitous as a means for explaining the Stroop phenomenon.

## Pitfall 1: General Definition of Conflict and Non-Conflict Stimuli

In the absence of a definition for the basic term, “conflict,” the control approach considers the Stroop stimulus as representative of all multidimensional stimuli. However, all multidimensional stimuli are not also conflict or Stroop stimuli. As we recounted, badly missing is the distinction between Stroop and non-Stroop stimuli. The missing distinction is conductive to the absurd notion that the ink-color response “green” to the word RED in *green* is comparable to the ink-color response “green” to a triangle in *green*. The missing distinction similarly leads to the notion that these ink-color responses are on the same foot as categorization responses to the word TABLE. Control theory holds that whenever there are multiple alternative responses to the (multidimensional) stimulus, there is conflict (in need of control). This idea, however, ignores the nature of the relations between the alternatives. The alternatives can be conflicting or *matching* as they are in Stroop-congruent stimuli (e.g., RED in red) *or* non-conflicting and non-opposing or simply logically unrelated. Stroop stimuli belong in the first class, but other multidimensional stimuli belong in the second class. Control studies blur the all-important dividing line between Stroop and non-Stroop stimuli.

What is the one property telling Stroop and non-Stroop stimuli apart? The defining feature of all Stroop stimuli is the existence of a *logical* relationship, compatibility or incompatibility, between their components. Each and every Stroop stimulus falls into one of the mutually exclusive and exhaustive classes of congruent or incongruent combinations. For example, all conceivable combinations of a color word and a print color *must* result in either a congruent (the word naming its color) or an incongruent (word and color mismatch) stimulus. Precluded is any other type of combination. By contrast, there is no logical conflict between the shape and the color of a green triangle. Again, an adequate theory of the Stroop effect must entail the uniqueness of Stroop stimuli as well as their distinct processing.

A ready example highlighting the last point is the so-called “emotional Stroop effect” (e.g., Algom et al., 2004, 2009). The emotional Stroop effect is the difference in color-naming performance between emotional (e.g., the word DEATH printed in red) and neutral (e.g., the word DOOR printed in red) stimuli. Because the words are not color words, these stimuli lack the logical relationship of conflict or correspondence between their attributes. The word DISEASE printed in blue is neither more nor less congruent than the word LECTURE presented in pink. The stimuli in the emotional Stroop task do not divide into congruent and incongruent combinations. Consequently, the Stroop effect cannot be calculated in studies of the emotional Stroop effect. Given a color-naming task, as in the classic Stroop task, the word BLUE printed in yellow (or in blue) is a Stroop stimulus, but the word CANCER printed in yellow (or in any other color) is not a Stroop stimulus. Conflict resides in the first type of stimuli but not in the second type of stimuli. Note that color naming may nonetheless be slower to CANCER than to TABLE, but that slowdown is not a Stroop effect. Clearly, all differences in performance do not derive from conflict.

## Pitfall 2: The Stroop Effect: Conflict and Facilitation

The control approach (as a Stroop theory) fails to account for Stroop facilitation. The standard Stroop experiment includes three types of stimuli: congruent stimuli (e.g., the word RED in red), incongruent stimuli (RED in green), and neutral stimuli (e.g., TABLE in red). The following equation defines the Stroop effect in all experimental designs:

$$\mathrm{Stroop}\;\mathrm{effect}=MRT\left(\mathrm{incongruent}\right)-MRT\left(\mathrm{congruent}\right)\text{,}$$

where MRT is the mean reaction time (RT) to name the ink color. The Stroop effect can be partitioned into Stroop interference (SI), so that SI = MRT (incongruent) – MRT (neutral), and Stroop facilitation (SF), so that SF = MRT (neutral) – MRT (congruent). Therefore, the Stroop effect equals the simple algebraic sum of interference and facilitation,

Stroopeffect=SI+SF

Note that the congruent stimulus “RED in red” does not entail any conflict, yet it is often a major contributor to the Stroop effect. People usually respond “red” to “RED in red” faster than they respond “red” to “TABLE in red”(=SF), and this facilitation enhances the observed Stroop effect. The Stroop effect is not equivalent to interference and conflict. It is also possible that the entire Stroop effect is produced by facilitation (e.g., Eidels et al., 2010; Eidels, 2012). A recognized theory of the Stroop effect, Tectonic theory (Melara and Algom, 2003), ascribes a major part of the Stroop effect to facilitation (rather than to interference).

It is worth pausing for a moment on the extreme theoretical version developed by Eidels (2012; see also Eidels et al., 2010). Eidels shows that a behavioral Stroop effect can derive from *independent* processing of the word and the color (i.e., there is an independent horse race between the processing channels). In Eidels’ theory, the color horse does not know the position, speed, or, indeed, the very existence of the word horse. Eidels (2012) uses stochastic modeling based on the following simple idea: For congruent stimuli, both processing channels (word, color) count for the same (correct) response, whereas for incongruent stimuli, only the color channel does. For example, for the congruent stimulus, RED in red, the fastest channel wins the race producing the correct response for the experimenter, regardless if it comes from the color (correctly) or from the word (incorrectly, but undetectably). Again, processing is completely independent. If so, there cannot be interference (or facilitation) simply because there does not exist any cross-talk between the processing channels. The notion of control and conflict is gratuitous in Eidels’ theory.

Ignoring theory, our main point is that merely observing a Stroop effect does not reveal the ingredients of interference and facilitation. Partitioning the effect by including the baseline condition of neutral stimuli is essential for arguing the case of conflict. In this respect, the majority of control studies of the Stroop effect did not include a baseline. Consequently, the Stroop effect cannot serve as a pure assay of conflict and control because the effect entails a significant non-conflict (i.e., facilitation) component. As a result, control cannot serve as a (parsimonious) theory of the Stroop effect.

## Pitfall 3: Physical Determinants of the Stroop Effect: The Relative Discriminability of the Words and the Colors

A major determinant of the Stroop effect is the relative salience or discriminability of the different words and ink colors used. When dimensional discriminability is matched, the time and accuracy needed to tell apart the words from one another is the same as the time and accuracy needed to tell apart the ink colors from one another. However, mismatched discriminability favoring words was present in virtually all control studies of the Stroop effect. Without dedicated preparation of the stimulus (not implemented in control studies), it takes participants longer to tell apart the ink colors from one another (e.g., red from green) than the words from one another (e.g., RED from GREEN). The presence of this asymmetry is critical because the more discriminable dimension disrupts performance on the less discriminable dimension (Sabri et al., 2001). Consequently, the task-irrelevant words affect performance with the ink colors (=Stroop effect) not because word reading is the habitual response (which generates conflict), but simply because the words differ perceptually from one another more than do the colors from one another. This factor of relative dimensional salience has been ignored in the control literature with serious consequences for Stroop theory.

**Figure 1:**
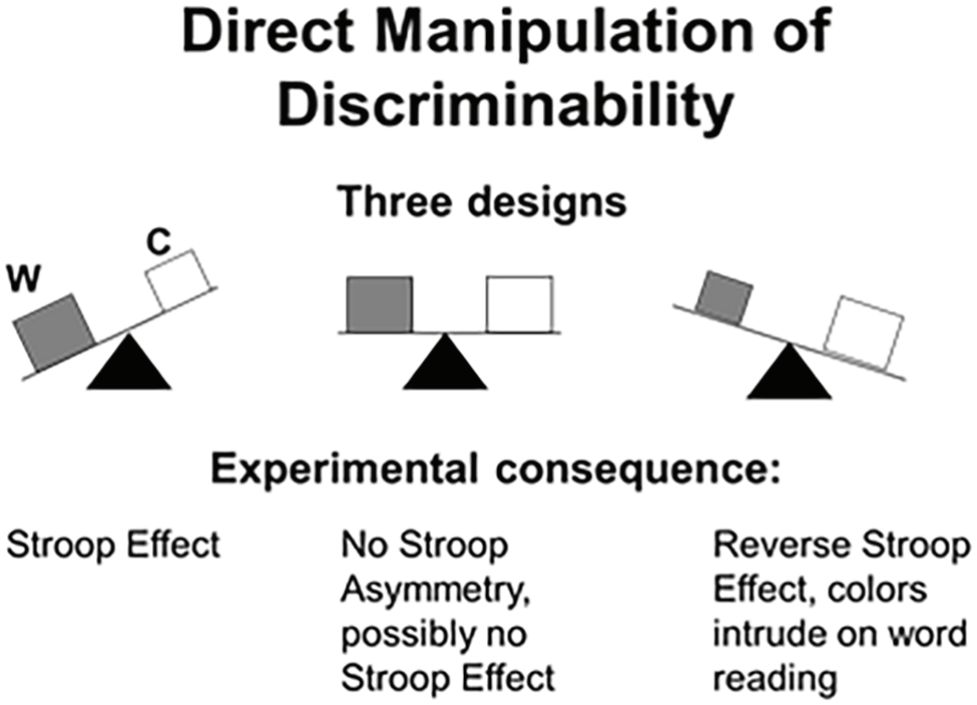
Schematics of the influence of relative salience on the outcome of the Stroop experiment. (Left-hand panel) The words (W) are more discriminable than the ink colors (C), the default setup in control studies. As a result, the irrelevant words intrude on color naming, thereby generating the Stroop effect. (Middle panel) The word and the colors are matched in discriminability, resulting in the elimination of the Stroop asymmetry in interference favoring words. (Right-hand panel) The colors are more discriminable than the words, so that word reading is now subject to interference from the ink colors more than vice versa (= reverse Stroop effect).

To recap, when the words are more salient than the colors (the default Stroop setup in the control literature), the usual Stroop effect appears. However, when the dimensions are made equally discriminable (by presenting appropriately matched values), the Stroop effect collapses. And, when the ink colors are made purposely more salient than the carrier words, a reverse Stroop effect emerges by which the ink colors intrude on word reading. We hasten to add that manipulations of salience entail nothing more than slight adjustment of the fonts (e.g., size, shape) and the colors (intensity, focality); they do not affect legibility or identification. Experimenters were able to produce a Stroop effect and a reverse Stroop effect or to eliminate the effect altogether at will (Garner and Felfoldy, 1970; Pomerantz, 1983; Melara and Mounts, 1993; Algom et al., 1996; Pansky and Algom, 1999, 2002; Sabri et al., 2001; Fitousi and Algom, 2006; Fitousi et al., 2009). A schematic summary of these results is provided in Figure 1.

**Figure 2:**
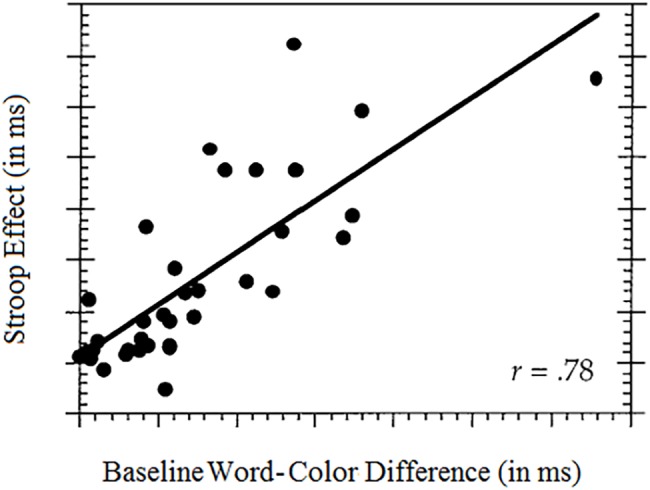
The influence of stimulus makeup on the Stroop effect: the larger the baseline word-color difference in salience (favoring word), the larger the Stroop effect.

The vital role of relative salience was discovered in a seminal work by Garner and Felfoldy (1970). More recently, Melara and Algom (2003) culled a sample of 35 published results from the Stroop literature and examined the relation between the *Stroop effect*, on the one hand, and the difference in *baseline salience* between word and color, on the other hand. The color Baseline task measures pure color performance: neutral words (e.g., TABLE, STREET, and CLOCK) in different colors are presented for color identification. The word Baseline task measures pure word-reading performance: Color words in uniform black are presented for word identification. Performance in these Baseline tasks can be compared to assess the ease or difficulty of classification along each dimension. Note that the Baseline tasks are *non-conflict* tasks in which the stimuli are one-dimensional. The Pearson correlation found between the word-color difference at baseline and the Stroop effect amounted to 0.78. This means that well over half of the variance in published values of the Stroop effect derives from mismatched salience between word and color. This relation is illustrated in Figure 2.

The effect of relative dimensional salience is evident already in Stroop’s classic study (Stroop, 1935). Stroop’s participants named the colors of 100 squares (pure color condition) in 63.3 s, on average, but read 100 words in black (pure word condition) in 41 s, on average – a staggering 22 s mismatch in task difficulty favoring words. When Stroop combined the two dimensions to produce color-word stimuli, *word* reading remained almost the same as in the pure word condition (mean of 43 s), but *color* naming was worse in the combined condition than in the pure color condition (mean of 110 s). The literature focused on this asymmetry in interference rather than on the prior asymmetry in baseline performance. However, given the summary of Figure 1, it is the latter that produced the former. Stroop’s results thus form a special case of the law by which the more salient dimension intrudes on the less salient dimension more than vice versa.

## Implications for the Control Approach

The results obtained with respect to the factor of relative salience are devastating for a control-based explanation of the Stroop effect. Conflict and control are said to depend on the number of conflict stimuli presented, those that produce the Stroop effect. In contrast to this notion, the literature shows that the Stroop effect can differ dramatically even when the number of conflict stimuli is kept constant. The Stroop result depends critically on the input-driven feature of word-color salience – with the same number of conflict stimuli presented. The condition entailing equal discriminability of word and color (Figure 1, middle panel) is particularly notable. In this condition, word and color are of equal salience, so that the typical perceptual advantage favoring the word dimension is removed. Despite the presence of a large number of conflict stimuli, the Stroop effect evaporates. In summary, the overall Stroop results mandate a stimulus-driven explanation. When the nominally irrelevant dimension (word) is more salient than the target dimension (color), attention to the color is compromised and expressed as the Stroop effect. However, this result is neither robust nor inevitable (Dishon-Berkovits and Algom, 2000; Melara and Algom, 2003). The upshot is that control cannot serve as a viable explanation of the Stroop effect.

## Pitfall 4: Color-Word Correlation and Word-Response Contingency Render Central Control Gratuitous

Another major factor affecting the Stroop effect is the number of congruent and incongruent stimuli included in the set. Any imbalance in the respective frequencies introduces a color-word correlation over the experimental presentations. This contextual effect has been attributed to conflict and control. By contrast, we show that the effect is data driven. Let us note that virtually all Stroop studies in the literature entail a biased design in the sense that there is a difference in the frequency of congruent and incongruent stimuli – so that the study entails a color-word correlation. The presence of this correlation renders the nominally irrelevant word predictive of the target ink color. On a trial, first noticing the word provides the participant a greater than chance probability of guessing the to-be reported color. By attending to the irrelevant word, the participant thus maximizes color performance. Because the Stroop effect gauges the influence of the irrelevant word (if there is no such influence, the Stroop effect is zero), a large color-word correlation encourages attention to the word, thereby producing a large Stroop effect. Notably, this large Stroop effect is generated by data-driven correlation, not by central control.

**Figure 3:**
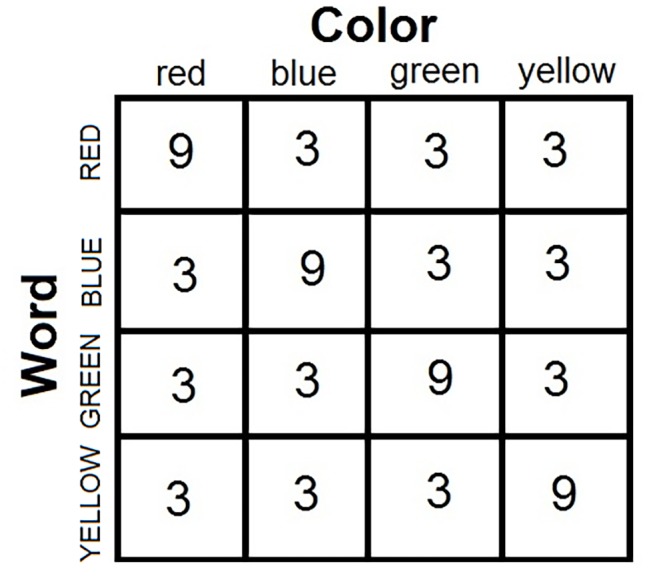
Anatomy of the standard Stroop experiment: Four color words are combined factorially with four ink colors to produce 16 color-words combinations. The entries are frequencies of presentations in 72 trials in the typical “balanced” experiment where trials in the congruent and incongruent conditions occur with equal frequency (36 congruent stimuli and 36 incongruent stimuli). The four combinations on the minor diagonal are congruent stimuli, whereas the 12 off-diagonal combinations are incongruent stimuli. The only way to equate the frequency of congruent and incongruent stimuli in the experimental block – the popular practice – is to present each congruent stimulus more often than each incongruent stimulus (in this case, three times as often). This design creates a correlation over the experimental trials between the nominally irrelevant words and the target ink colors.

It might come as a surprise to realize that biased designs are used in the vast majority of published Stroop studies. Consider the standard and most popular Stroop design in the literature. Four color words are combined with the corresponding four colors in a factorial design to yield the basic matrix of 16 color-word stimuli (see Figure 3). Of these 16 stimuli, four are congruent (in the diagonal of the matrix) and 12 are incongruent (off diagonal). In the face of this asymmetry, investigators typically present an equal number of congruent and incongruent stimuli in the experimental block. The typical block thus includes 36 congruent and 36 incongruent stimuli. Note that this parity is *only* possible by presenting each congruent stimulus more often the each incongruent stimulus. In the popular design, each congruent stimulus is presented nine times, whereas each incongruent is presented three times to create the matched frequency of 36 presentations. The *a priori* probability of a color given a word is not equal across all colors, so that the word becomes predictive of the target color. A color-word correlation thus is created in this standard Stroop design.

**Figure 4:**
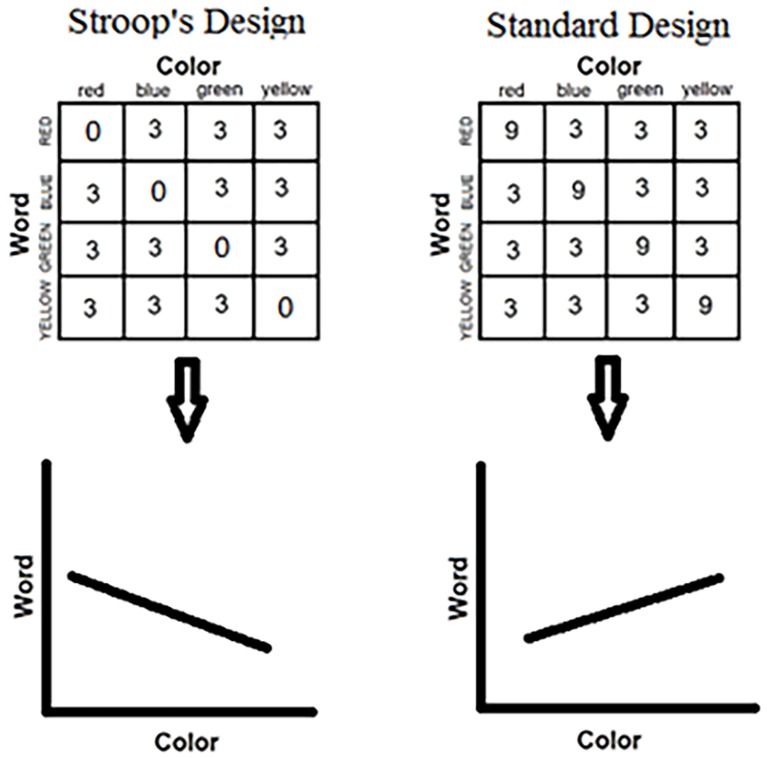
Allocation of colors to words to form the set of color-word stimuli in two experimental situations. The left-hand panel depicts a “negative” correlation, in which only incongruent stimuli are included in the set. This was Stroop’s experimental design in his original study (Stroop, 1935). The negative slope of the regression line illustrates the fact that one dimension is predictive of the other. The right-hand panel depicts a “positive” correlation, in which the conditional probability of a color (word) given a word (color) is greatest for the congruent combinations. This predictive relation is illustrated by the positive slope of the regression line. This relation lurks in the standard most popular Stroop design in the literature.

In point of fact, biased Stroop designs started with Stroop himself (Stroop, 1935). In his experimental block, Stroop used only incongruent stimuli. None of the color words appeared in its own color. Unwittingly, Stroop introduced a correlation between words and colors in his list. Noticing first that the word was RED, the participant could safely infer that the ink color is not red. A sizable correlation was thus created, which, in turn, generated the large Stroop effect observed (see Figure 4).

**Figure 5:**
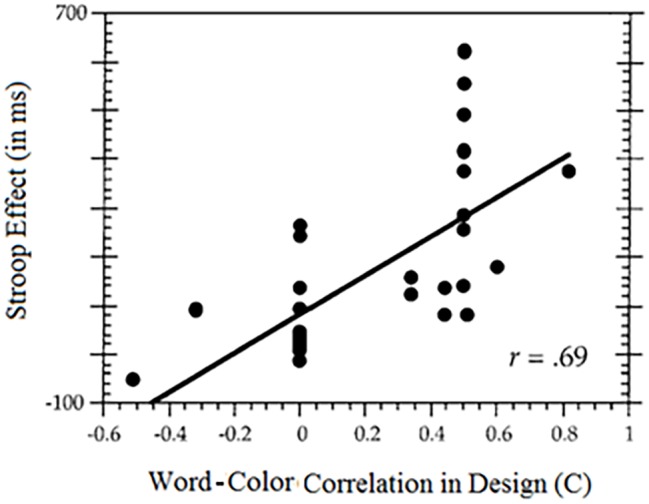
The relation between the color-word correlation built into the experimental design, usually by unequal presentation of congruent and incongruent stimuli (measured by the contingency coefficient, C) and the Stroop effect. The larger the correlation built into the design, the larger the Stroop effect.

In an effort to estimate the influence on the Stroop effect of word-color correlation, Melara and Algom (2003) calculated the correlations lurking in the designs of 35 experiments from the literature. They plotted the Stroop effect against the built-in correlation in the design. The results are noteworthy: the correlation between the Stroop effect and the word-color contingency in the design amounted to 0.69. This means that close to 50% of the variability in the published Stroop effects is attributable to the word-color correlation built into the design of the experiment (Figure 5).

If a built-in correlation exists in most standard Stroop studies, the correlation is even more marked and extreme in control studies. As we just recounted, the standard 50–50% congruency design (with four colors and four color words) already entails an appreciable correlation between the words and the colors. The grossly imbalanced congruency structure created in control studies produces an even larger color-word correlation. The common design in control studies typically entails 80% (in)congruent stimuli, which translates to a sizeable color-word correlation. Perception of this correlation suffices to explain the results.

The upshot is that the notion of fine grain, centrally imposed control is gratuitous when explaining the Stroop effect. When a correlation makes the words predictive of the colors, people attend to the word, so that exclusive attention to the color is compromised – and a large Stroop effect emerges. People are eminently sensitive to correlations between stimuli in their environment, and the Stroop effect is a manifestation of this sensitivity (Kareev, 2000).

## Directional Proportion-Congruity (PC) Effects

Proponents of control or conflict point to the directional effects observed in biased designs: the larger the proportion of incongruent stimuli in the set, the smaller the Stroop effect. At first glance, color-word correlation cannot generate this asymmetric outcome (the PC effect). The PC effect is a major source of evidence presented in support of the control and conflict monitoring account of the Stroop effect. On close scrutiny tough, the PC effect results from a correlation between specific *words* and specific *responses* in the experiment. In all 2 (word) × 2 (color) designs or in designs in which incongruent stimuli come in a favored color (e.g., the word RED comes mostly in green), the larger the relative number of incongruent stimuli, the larger the correlation between a given word and a given response. This relation is termed the *contingency-learning* account of Stroop and PC effects (Schmidt and Besner, 2008; Schmidt, 2016a,b, 2019; Schmidt et al., 2018). The contingency account readily explains the PC effect:

… in the mostly congruent condition, words are presented most often in their congruent color (e.g., RED 75% of the time in red). As such, color words are strongly predictive of the congruent response, which benefits congruent trials. On incongruent trials (e.g., RED in green), however, the word mispredicts the color response, resulting in a cost. The net result is an increased Stroop effect. In the mostly incongruent condition, the situation is reversed. Depending on the exact manipulation, color words might be presented most often in a specific incongruent color (e.g., GREEN most often in red). Thus, words are accurately predictive of the *incongruent* response, and mispredict a congruent response. The net effect is a reduced congruency Stroop effect. *What is most interesting about the contingency learning account of the PC effect is that it is unrelated to conflict, control…* [On this account], *learning of stimulus–response correspondences is all that matters.* (Schmidt, 2016a, p. 1, emphasis added)

Schmidt’s stimulus-driven account shows that the correlation created in biased Stroop designs between the words and the (color) responses readily explains the PC effects, which are otherwise attributed to conflict and control. Applying Occam’s razor, Schmidt’s account is favored over the central control account. We should mention that in general contingency learning is not related to attention per se. However, it is an important contextual factor within the Stroop domain (after all, Stroop is a test of selective attention). Within the Stroop task, contingency affects the selectivity of attention to the stimulus attributes, hence the magnitude of the Stroop effect observed.

### Are Color-Word Correlation and Word-Response Contingency Both Necessary?

The *color-word* correlation account by Melara and Algom (2003) and the *word-response* contingency account by Schmidt (2019) explain variations in the magnitude of the Stroop effect without any reference to the notions of control and conflict adaptation. The two accounts actually complement each other. On both views, the Stroop effect is the result of perception of correlation or contingency in the data (see also Lorentz et al., 2016). The correlation and contingency accounts rest on a common principle, but a word seems in order to clarify their distinct roles in the Stroop domain.

Contingency learning best explains the PC effects observed in 2 (word) × 2 (color) designs and in multi-valued designs with favorite pairings of incongruent stimuli. Color-word correlation readily explains the Stroop results obtained in the standard 4 (word) × 4 (color) designs that do not include favorite incongruent pairings. This account also explains the appearance of the Stroop effect in so-called balanced designs entailing 50–50% of congruent and incongruent stimuli. In the study by Dishon-Berkovits and Algom (2000), incongruent stimuli appeared only once under some conditions (so that contingency learning was impossible), yet the authors showed how color-word correlation produced their results in this unusual matrix. In summary, both the correlation and the contingency varieties are useful in accounting for Stroop results. Significantly, they do so without appeal to central control, conflict, or conflict adaptation.

## The Gratton Effect

As we recounted at the outset, the Gratton effect (Gratton et al., 1992) or more appropriately, the Congruency Sequence effect (Schmidt, 2013, 2019; Weissman et al., 2014), comprises arguably the strongest piece of evidence marshaled in support of the conflict monitoring account. To reconstruct the chronology, the original finding by Gratton and her colleagues (Gratton et al., 1992) has lain dormant for almost a decade when it was resuscitated and brought to the fore by Botvinick et al. (2001) to support their newly formed theory of central conflict monitoring. Since the publication of the Botvinick et al. model, research on the Gratton effect has intensified appreciably, sustaining a vigorous debate on the source of the effect: genuine on-line conflict monitoring or yet another trial-sequence-based facilitation (e.g., Effler, 1978; MacLeod, 1991). Given the role of the Gratton effect in deciding the fate of the conflict-monitoring model as a Stroop theory, we devote some space to elucidate the ongoing debate.

The Gratton effect is the sequential variation by which the RT to a Stroop-incongruent stimulus is faster after experiencing another Stroop-incongruent stimulus than after experiencing a Stroop-congruent stimulus (e.g., Mordkoff, 2012; Weissman et al., 2014; Schmidt, 2019). Less attention has been given to the parallel observation that RT to a Stroop-congruent stimulus is usually faster after experiencing another Stroop-congruent stimulus than after experiencing a Stroop-incongruent stimulus (e.g., Mayr et al., 2003). This latter observation alone should have cast doubts on the validity of the conflict monitoring model as a Stroop theory. After all, congruent-congruent sequences do not entail (high) conflict, yet these sequences affect Stroop performance to the same extent as do incongruent-incongruent sequences. The possibility that *both* types of sequences are accounted by factors unrelated to conflict becomes all the more likely. The focus on incongruent-incongruent sequences in the literature comes from the theoretical stress on conflict and its on-line resolution. On that view, the role of fine-grain central control during Stroop performance is to enhance target (color) processing and reduce task-irrelevant (word) processing on a *trial-by-trial* basis. It is these top-down penetrations that produce the Gratton effect: experiencing conflict instantly triggers control activity, which results in better performance on the immediately following trial.

## The Mayr et al. Challenge

Barely a year after the formal development of the central-conflict-monitoring model (Botvinick et al., 2001), Mayr et al. (2003) challenged the ability of the model to provide a valid account of the Gratton effect. In their seminal study, Mayr et al. (2003) pinpointed correctly a central (if implicit at that point) assumption of the conflict monitoring model: The conflict that regulates performance is *stimulus-independent*. According to the conflict monitoring model, the incongruent-incongruent sequence of RED in green-RED in green (complete repetition) should produce the same adaptation as the incongruent-incongruent sequence of RED in green-BLUE in yellow (complete change). According to conflict monitoring theory, it is the conflict that counts, not the means of generating it. Mayr et al. (2003) have shown in contrast that the Gratton effect is profoundly stimulus dependent.

Mayr et al. (2003) used the flanker task [2(targets) × 2(flankers)], noting that complete repetitions comprise 50% of the incongruent-incongruent sequences in any standard flanker task (as do 50% of the congruent-congruent sequences). They recorded the typical Gratton effect in their experiment. However, when the authors examined their data separately for sequences of complete repetition and sequences entailing change, they found the Gratton effect only for the former. Mayr et al. (2003) concluded that “stimulus specific repetition … can provide a complete explanation of the … pattern observed” (p. 451). The authors then conceived a second flanker experiment where immediate complete repetitions were eliminated altogether and where response repetitions were also eliminated (by presenting the flanker display horizontally or vertically on alternate trials and requiring appropriate left-right or up-down responses). Note that the absence of repetitions is irrelevant for the conflict monitoring account, but it is critical for accounts based on input-driven processes (in particular, on priming of complete repetitions). The latter account predicts that eliminating repetitions should eliminate the Gratton effect. Consistent with this prediction, no Gratton effect was observed in Mayr et al.’s (2003) second experiment.

Mayr et al. (2003) noticed a further feature of the data that was inconsistent with the conflict monitoring account. Although immediate repetitions were avoided in their second experiment, such repetitions could and did occur between trial *n*−2 and trial *n*. Stimulus-driven accounts predict that an attenuated Gratton effect should still appear on such trial *n*−2 to trial *n* repetitions. The conflict monitoring account, by contrast, lacks a mechanism that allows for adaptation to occur across non-conflicting intermediate trials. The results disconfirmed the central-control model, showing instead the presence of adaptation across non-adjacent repetitions. Mayr et al. (2003) stated in their conclusion that “conflict-triggered control is not necessary to explain the [Gratton] effect” (p. 452), that “regulative demands are bypassed by stimulus-driven repetitions” (p. 452), thereby justifying their title on the presence of the Gratton effect “in the absence of executive control.”

## Recent Gratton Research

Mayr et al.’s (2003) formative study heavily impacted Gratton research in the ensuing two decades (see Schmidt, 2019, for a review of this research). The Mayr et al. (2003) study made it clear that the standard 2 (targets) × 2 (flankers) flanker task is hopelessly biased by stimulus-stimulus and stimulus-response correlations. The same confounds apply to the Simon task (Simon, 1969; Simon and Berbaum, 1990; see also Hatukai and Algom, 2017) and to the small-set version [2 (words) × (colors)] of the Stroop task. To remove the biases from the Stroop-, Simon-, and the flanker-task (by far the most popular test used), succeeding investigators applied both of Mayr et al.’s (2003) strategies: statistical and experimental. The first approach allows for stimulus repetitions (complete or of component features) to occur but removes them statistically in subsequent analysis (e.g., Schmidt and De Houwer, 2011; see also Mordkoff, 2012). In the second approach, stimulus and response repetitions are not presented or allowed in the experiment itself. To exclude repetitions from the experimental design, most researchers employed Mayr et al.’s (2003) alternate horizontal-vertical procedure, often extending the flanker design in time (e.g., Schmidt and Weissman, 2014). The overall results obtained (in both approaches) do not support the conflict monitoring account.

Because our goal in this critique is *conceptual* scrutiny, we next highlight just a few important points (again, see Schmidt, 2019, for a detailed review of recent research). The goal of studies adopting the second “experimental approach” was to test the presence of the Gratton effect under sterile, confound-free stimulus conditions. If the Gratton effect still emerges under such conditions, the central control account is bestowed powerful support. Consequently, strenuous attempts have been made to purge all species of stimulus- and response-based contingencies from the experiment. Unfortunately, the elimination of the confounds came at the cost of eliminating the flanker task itself, i.e., deforming it in a significant way. The popular tactic has been using Mayr et al.’s (2003) horizontal-vertical alternation and extending the task in time, so that the target display is preceded by an advance cue (e.g., Kunde, and Wühr, 2006; Schmidt and Weissman, 2014; Weissman et al., 2014). However, this tactic likely compromised the nature of the flanker task as an interference design, so that the results obtained probably hinged on the perceived validity of the advance cue. We note in parenthesis that the alternation procedure itself might invite unrelated processes into the experiment (e.g., benefits/costs of switching; see also, Schmidt and De Houwer, 2011). It is moot whether the “Gratton effect” observed in such temporal prime-probe tasks is truly comparable with the original effect observed in the standard flanker task. The following Gedanken experiment can clarify this issue, i.e., how the “Gratton effect” can be observed in the absence of conflict or interference.

Suppose that the target display is a shape in color and that the task is to name the color. On different trials, the shape can be a triangle or a circle and its color can be red or green. Suppose further that the display is preceded by a prime, a patch of red or green color. Clearly, a red triangle is not a conflict stimulus, yet a spurious “Gratton effect” may well be observed in this conflict-free task. The prime-probe experiments in the literature, while tightly controlled for stimulus and response confounds, might not comprise a real test of the source of the Gratton effect. The results obtained in the confound-free, prime-probe, and temporal flaker experiments are commensurably mixed and difficult to interpret. Some studies reported the Gratton effect (e.g., Schmidt and Weissman, 2014; Weissman et al., 2014), but further features of the results are difficult to interpret and are certainly inconsistent with a conflict monitoring account. For example, Weissman et al. (2014) did not find a correlation between the Gratton effect and the flanker effect and have sometimes recorded a negative Graton effect (a larger flanker effect after incongruent-incongruent sequences). Note that a negative Gratton effect is impossible under conflict monitoring.

Considering the Stroop effect itself, methodological problems have been plaguing that research, too. Following the Mayr et al. (2003) study, the 2 (words) × 2 (colors) task is no longer feasible due to the stimulus and response correlations inhering in this design. The popular 4 (words) × 4 (colors) design (see Figure 2) obviously is more appropriate, but there exists the problem of the relative number of congruent stimuli. As we shown, the popular 50%–50% congruent-incongruent ratio entails a sizeable correlation, biasing performance (Dishon-Berkovits and Algom, 2000; Melara and Algom, 2003; Schmidt and Besner, 2008). Only a truly random allocation of the colors to the words can eliminate this bias. Random combinations in a 4 × 4 design entail a rate of 25% congruent stimuli. However, even this regime is open to further biases related to stimulus sequences. Removing all confounds from the Stroop task (if at all possible) remains a daunting task (Mordkoff, 2012; see also Sabri et al., 2001; Melara and Algom, 2003; Hommel et al., 2004; Schmidt and De Houwer, 2011). Existing research did not match those exacting standards. For example, Weissman et al. (2014) used four color words and four colors but paired each word with only two of the colors. The study by Mayr and Awh (2009) came close with the authors using a large set of 6 (words) × 6 (colors) and changing the rate of congruent stimuli across separate blocks of the Stroop task. The block with lowest rate included 30% congruent stimuli, a figure which still deviated appreciably from random allocation (the full matrix of 36 color-word combinations includes six congruent stimuli or 17%, not 30%; see also Schmidt and De Houwer, 2011). The problems granted, most important for the present concerns is the uniform absence of adaptation or the Gratton effect in the classic Stroop task, a consistent result in studies using either the statistical approach or the experimental approach [we should mention that Duthoo et al. (2014) recorded the Gratton effect in their Stroop tasks, but, again, the control against biases was less than compelling].

We conclude with four final observations. First, the hallmark of modern Gratton research is the stimulus dependence of adaptation. Minor changes in preparation and paradigm can determine the presence or magnitude of the Gratton effect. For example, in prime-probe studies, the spatial location of the prime and the probe (same, different) greatly affects the outcome. In a similar vein, stimulus overlap and response overlap in cross-task Gratton studies are a major determinant of adaptation. These observations violate the basic assumption of the conflict monitoring account on the *stimulus-independence* of adaptation. Second, another basic (if unarticulated) assumption of conflict monitoring is that adaptation is *task-independent*. In violation of this assumption, recent research has shown that adaptation is singularly task-dependent. The Gratton effect can be observed in the Simon task but not in the Stroop or in the flanker task using the same design within the same study (Weissman et al., 2014). Conflict adaptation typically does not generalize across tasks. And, when conflict in the Stroop task results in adaptation on the next conflict trial in the Simon task, the transfer is typically explained by shared features and task sources. Third, the observation that congruent-congruent sequences produce the same result as incongruent-incongruent sequences implies that the Gratton effect is not related to conflict. Our fourth and final observation is methodological. Extant Gratton research treats “interference tasks” such as those of Stroop, Simon, and flanker on the same footing. However, all interferences or conflict tasks are not the same (Chajut et al., 2009). Thus, the flanker and Simon tasks entail spatial attention, with targets and distractors separated in space. The Stroop task, by contrast, does not entail spatial attention: The color and the word occupy the same location in space, so that space-based attention to isolate the target is impossible. In the Stroop task, people dissect mentally the stimulus object in order to respond to the task-relevant feature.

On balance, the available evidence with regard to the Stroop or Gratton effect is inconsistent with the theory of centrally guided conflict monitoring account. Instead, it is local, input-driven bottom-up processes that likely generate the Gratton phenomenon (when it is observed). It is important to bear in mind that there is in fact a long history of research on sequential effects in the Stroop task. Dalrymple-Alford and Budayr (1966) may have been the first authors to report such effects more than half of century ago. In subsequent research, a fair number of sequential effects have been documented, some entailing interference and some, like the Gratton effect, facilitation (see MacLeod, 1991, for a review). Notably, none of the authors associated with the various effects thought it necessary to evoke the heavy machinery of centrally controlled conflict management as an explanatory device. Given the variety of sequential effects identified within basic Stroop research, the reader may well perceive that there is something not altogether satisfactory about the disproportionate exposure and study of a single facilitatory effect. The reason (not justification) for that one-sided research is obvious: the Gratton effect has been imported to a theory and domain, which, at its roots, is foreign to the Stroop effect.

## Conclusion

Performance in the Stroop task and the resulting Stroop effect does not seem to involve higher-order cognitive level processes of control, nor does it seem likely that minute top-down penetrations determine responding in the Stroop and allied tasks. The particular theoretical embodiment assuming such trial-by-trial top-down penetrations, the account called conflict monitoring, is not optimally suited to explain the gamut of results obtained over the years in the vast Stroop literature. The conflict monitoring account even does not recognize the existence of major Stroop variables apart from the duo of the PC and Gratton effects (see MacLeod, 1991 and Melara and Algom, 2003, for reviews of Stroop research). Focusing solely on that pair of effects, most monitoring studies are compromised by the input-based confounds noted. The few confound-free studies that did demonstrate adaptation (most did not) – allegedly supporting central control – ignored alternative input-based explanations, at once more plausible and parsimonious. We believe that the converging evidence provided by the findings reviewed in this article confirms the lawful dependence of the Stroop effect on input factors and seriously challenges centrally controlled conflict monitoring as a valid theory of the Stroop effect. All facets of the effect are explained in a straightforward fashion by input-driven selective attention (indeed, its failure). Concerning the PC and Gratton effects in particular, all that is truly involved is perception of color-word correlation and of word-response contingency.

**Figure 6:**
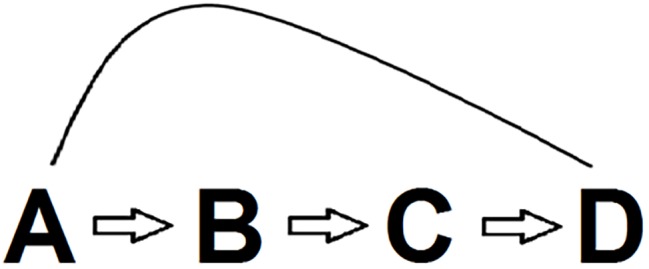
Possible chain of reasoning accommodating both the basic Stroop findings reviewed in the paper and the conflict monitoring and control account. Briefly, basic Stroop variables (A) drive conflict (B), which, in turn, drives control (C), so that they produce (D) the Stroop outcome, including PC and Gratton effects. The conflict monitoring model basically entails that B and C produce D. However, since it is possible to get directly from A to D, the conflict monitoring model is gratuitous as a Stroop theory.

This much granted, we realize that conflict monitoring modelers (e.g., Yeung et al., 2011) may agree with the importance of the factors uncovered in basic Stroop research but maintain that conflict monitoring also plays a role in addition to these factors. This way of reasoning is depicted in Figure 6. Conflict monitoring theory basically entails that conflict (B) drives control (C) so that they produce the Stroop outcome including notably PC and Gratton effects (D). Monitoring modelers probably have no problems with the link between (A), the basic Stroop variables reviewed in this paper, and (B). At a first glance, the relation between (A) and (B), the primary theme of this review, might be regarded as orthogonal to the validity of the conflict monitoring account. However, the present review makes it eminently clear that one can get directly from (A) to (D), so that (B) and (C) are not needed. In other words, once one is willing to accept the principles learned from basic Stroop research, then conflict monitoring and control are superfluous added assumptions.

Of course, there is a trivial sense in which people willfully apply control over what they do and experience. They come to the lab as planned, they choose to perform with their eyes open, and they are in charge of many other perfunctory chores. In the Stroop task itself, people follow quite successfully the instructions to name the colors and ignore (overtly at the least) the words. Indeed, there are task-demand units already included in the computational model of Cohen et al. (1990). For example, in the study by Bauer and Besner (1997), the mental set espoused by the observer determined the Stroop outcome with the same stimuli and the same responses. We acknowledge of course these instances of control, but they do not serve (nor are they meant to serve) as a comprehensive theory of the Stroop effect.

Pursuant to the previous point, we also acknowledge that the control and conflict monitoring account include the notion of attention. However, “attention” in this model is a generic process, governed centrally (by a homunculus?), and, like “conflict,” is not rigorously defined. By contrast, attention as studied in the Stroop literature is a well-defined process of selectivity. It is concerned with determining the quality of focusing on the task relevant attribute while ignoring irrelevant information. The whole process is governed by bottom-up contextual factors.

Perhaps, also, there would be something instructive to be gained from the way that proponents of control theory come close to espousing the present view in certain cases. These researchers are just unable to jettison the underdefined concept of control even when clearly unwarranted to make their case. Thus, Julie Bugg, a leading investigator of control, proposed to classify the accounts of Stroop performance into *expectation-based* and strategically guided accounts versus *experience-based* and reactive adjustment accounts (e.g., Bugg et al., 2015). The latter class is comparable to the present approach, but then the authors hasten to add that “experience-based accounts also subsume conflict-monitoring accounts” (Bugg et al., 2015, p. 1350). The same indetermination marks Tom Braver’s influential model, the Dual Mechanisms of Control (DMC; Braver, 2012). Braver, a foremost researcher of control, proposes to distinguish between two species of control, “proactive control” and “reactive control.” The former acts strategically through top-down adjustments, whereas the latter acts locally in response to the stimulus that has just occurred. Concerning reactive control, Braver states that “[it] is stimulus driven and transient … is stimulus dependent … [and] is reliant on strong bottom-up … cues” (Braver, 2012, p. 108). Remove “control” from Braver’s depiction and you have the view that we are presenting here. The problem we noted is that there does not seem to be any process exempt from control in Braver’s (and in other proponents of control) view (thereby undermining the value of “control” as a useful scientific concept). Retaining “control” in all places and instances may be due to the peculiarity of these investigators’ disposition: associating each trifle mental act with a specific brain structure and activation (Braver, for one, claimed to pinpoint different loci and activation for proactive and reactive control). However, such activations have not been shown to be *uniquely* linked to a specific act or task, and, in any case, recording activation in brain loci does not *ipso facto* comprise a theory and explanation.

Our skeptical conclusions agree with those arrived by Schmidt (2019) and by Firestone (2013) and Firestone and Scholl (2016) in the general domain of alleged top-down influences in perception. To echo Firestone (2013), the deepest shortcoming of central conflict monitoring theory is not the lack of support in most available evidence, but that it is simply the wrong kind of theory for the Stroop effect that it has appropriated from input-driven attention.

## Author Contributions

Both authors contributed equally to the manuscript.

## Conflict of Interest Statement

The authors declare that the research was conducted in the absence of any commercial or financial relationships that could be construed as a potential conflict of interest.
